# The Swiss Multiple Sclerosis Cohort-Study (SMSC): A Prospective Swiss Wide Investigation of Key Phases in Disease Evolution and New Treatment Options

**DOI:** 10.1371/journal.pone.0152347

**Published:** 2016-03-31

**Authors:** Giulio Disanto, Pascal Benkert, Johannes Lorscheider, Stefanie Mueller, Jochen Vehoff, Chiara Zecca, Simon Ramseier, Lutz Achtnichts, Oliver Findling, Krassen Nedeltchev, Ernst-Wilhelm Radue, Till Sprenger, Christoph Stippich, Tobias Derfuss, Jean-François Louvion, Christian P. Kamm, Heinrich P. Mattle, Christoph Lotter, Renaud Du Pasquier, Myriam Schluep, Caroline Pot, Patrice H. Lalive, Özgür Yaldizli, Claudio Gobbi, Ludwig Kappos, Jens Kuhle, SMSC Scientific Board

**Affiliations:** 1 Department of Neurology, Regional Hospital Lugano (EOC), Lugano, Switzerland; 2 Clinical Trial Unit, University Hospital Basel, Switzerland; 3 Neurology, Departments of Medicine, Biomedicine and Clinical Research, University Hospital Basel, Basel, Switzerland; 4 Department of Neurology, Cantonal Hospital St. Gallen, St. Gallen, Switzerland; 5 Department of Neurology, Cantonal Hospital Aarau, Switzerland; 6 Medical Image Analysis Centre, University of Basel, Basel, Switzerland; 7 Neuroradiology, Department of Radiology, University Hospital Basel, Basel, Switzerland; 8 RodanoTech, Geneva, Switzerland; 9 Department of Neurology, Inselspital, Bern University Hospital and University of Bern, Bern, Switzerland; 10 Swiss Multiple Sclerosis Society, Zürich, Switzerland; 11 Department of Neurology, University Hospital of Lausanne (CHUV), Lausanne, Switzerland; 12 Department of Neurology, University Hospital of Geneva (HUG), Geneva; National Institutes of Health, UNITED STATES

## Abstract

The mechanisms leading to disability and the long-term efficacy and safety of disease modifying drugs (DMDs) in multiple sclerosis (MS) are unclear. We aimed at building a prospective cohort of MS patients with standardized collection of demographic, clinical, MRI data and body fluids that can be used to develop prognostic indicators and biomarkers of disease evolution and therapeutic response. The Swiss MS Cohort (SMSC) is a prospective observational study performed across seven Swiss MS centers including patients with MS, clinically isolated syndrome (CIS), radiologically isolated syndrome or neuromyelitis optica. Neurological and radiological assessments and biological samples are collected every 6–12 months. We recruited 872 patients (clinically isolated syndrome [CIS] 5.5%, relapsing-remitting MS [RRMS] 85.8%, primary progressive MS [PPMS] 3.5%, secondary progressive MS [SPMS] 5.2%) between June 2012 and July 2015. We performed 2,286 visits (median follow-up 398 days) and collected 2,274 serum, plasma and blood samples, 152 cerebrospinal fluid samples and 1,276 brain MRI scans. 158 relapses occurred and expanded disability status scale (EDSS) scores increased in PPMS, SPMS and RRMS patients experiencing relapses. Most RRMS patients were treated with fingolimod (33.4%), natalizumab (24.5%) or injectable DMDs (13.6%). The SMSC will provide relevant information regarding DMDs efficacy and safety and will serve as a comprehensive infrastructure available for nested research projects.

## Introduction

Multiple sclerosis (MS) is a complex demyelinating disease of the central nervous system (CNS) and represents the most common cause of acquired neurological disability in young adults in developed countries [1]. The prevalence of MS in Western Europe and North America is approximately 1/1,000, with an incidence of 5–10 new cases per 100,000 individuals every year [2]. The relatively early age of onset (approximately 30 years old) and the typically chronic and disabling course result in a substantial socio-economic burden [3, 4]. Approximately 10,000 individuals are thought to be affected by this condition in Switzerland.

Our understanding of MS aetiology has greatly improved in the last 10–20 years. We now know that a large number of genetic variants, together with several putative environmental agents (including serum vitamin D levels, Epstein-Barr virus infection and smoking) determine MS susceptibility [5, 6]. Immunological and pathological studies have provided evidence for an important role played by the immune system in the demyelinating process seen in MS [7, 8]. Neuronal degeneration is also key and becomes especially evident in the progressive phase of the disease, when brain atrophy and irreversible disability accumulation are more prominent [7, 9]. This increasing knowledge has provided the rationale behind the development of several disease modifying drugs (DMDs) in addition to the first generation of injectable DMDs (interferon β-1b, interferon β-1a and glatiramer acetate). These include natalizumab, fingolimod, dimethyl fumarate, teriflunomide and alemtuzumab [10–15].

Despite this, the mechanisms driving the occurrence of clinical relapses, disability accumulation and the passage from the relapsing-remitting to the secondary progressive phase of the disease are still elusive [16, 17]. In addition, although the number of available DMDs has increased, their efficacy and safety need long-term evaluation. This cannot be achieved in the traditional settings of clinical trials, which are usually limited in time and rarely designed as “head to head” trials comparing different DMDs. Furthermore, prognostic factors associated with response to treatments are lacking and their identification will be key to develop personalized treatment strategies. Finally, biological markers able to monitor subclinical pathology and treatment efficacy are lacking and strongly needed [18]. Long-term observational studies combining the systematic acquisition of clinical data, imaging and laboratory measures can help us respond to these unmet needs.

The Swiss MS Cohort-Study (SMSC) was initiated in 2010 and started recruiting in June 2012 with 4 specific aims. 1) To build and maintain a long-term cohort of MS patients in Switzerland. 2) To conduct a systematic follow-up of these MS patients with standardized collection of demographic, clinical, and MRI data as well as body fluids material. 3) To maintain and improve the high standard of care of MS patients in Switzerland and elsewhere by assessing the long term efficacy and safety profile of available DMDs for MS. 4) To build a comprehensive infrastructure available for nested projects aimed at developing prognostic indicators and biomarkers of disease evolution and therapeutic response.

## Materials and Methods

The SMSC is a prospective multicentre cohort study performed across seven Swiss centres: the Cantonal Hospital of Aarau, the University Hospitals of Basel, Berne, Geneva and Lausanne, the Regional Hospital of Lugano and the Cantonal Hospital of St. Gallen. The SMSC is an investigator initiated study and does not pursue financial interests. All participating centres contributed to the design of the SMSC and are part of the governing committee (the SMSC Scientific Advisory Board (SCB)). The Department of Neurology at the University Hospital Basel was designated as the coordinating centre. The SCB covers all aspects regarding conduct of scientific nested research projects within the SMSC. The SMSC received ethical approval by independent ethics committees (EC) at each participating centre (EC Aarau, EC Basel, EC Bern, EC Geneva, EC Lausanne, EC Lugano, EC St Gallen). The final goal is to follow-up approximately 1,000 MS patients for at least 5–10 years.

### Patient Recruitment and Inclusion Criteria

To be included in the study individuals need to be diagnosed with either relapsing-remitting MS (RRMS), secondary-progressive MS (SPMS) or primary-progressive MS (PPMS) according to the 2010 revised McDonald [19 or previous established diagnostic criteria (McDonald or Poser) [20, 21]. Patients diagnosed with a clinically isolated syndrome (CIS), radiologically isolated syndrome (RIS) or neuromyelitis optica (NMO) can also be included [22, 23]. We aimed to build a cohort of patients that was homogeneous and informative for the questions we wanted to answer. Eligible criteria were: 1) absence of treatment with any MS specific DMD, or 2) need to either begin or switch to a different DMD as judged by the treating physician, or 3) initiation of a new DMD or current treatment with natalizumab or fingolimod, or 4) switch to a different DMD. Patients are included in the SMSC only after a written informed consent is signed. Every effort is made to reduce drop-outs to a minimum. If a patient moves to another place within Switzerland, the original centre arranges a consultation with the nearest participating centre.

### Baseline and Follow-Up Clinical Data Collection

Demographic and clinical variables collected at baseline include sex, date of birth, ethnicity, family history of MS, pregnancy history, date of first MS symptom, date of second relapse, number of relapses in the last two years, date of diagnosis, date of start of progression (if applicable), current MS specific DMDs, concomitant medical conditions and medications. Standardized clinical assessments with functional system score and Expanded Disability Status Scale (EDSS) calculation are performed by certified raters (http://www.neurostatus.net/). Every patient is followed-up every 6 or 12 months +/-45 days as judged by the treating physician. The occurrence of relapses, disability progression (as measured by the EDSS), DMDs initiation or interruption, DMD related adverse events, additional medical conditions and concomitant medications are recorded at each visit. Laboratory measures and evoked potentials are also performed in a smaller fraction of patients.

### Body Fluids Collection

Collection protocols for serum/plasma and cerebrospinal fluid (CSF) have been published by consensus among 26 groups participating in the BioMS-eu network [24]. We apply these protocols to collect CSF and blood samples from SMSC patients. Collection of CSF samples is optional and limited to patients undergoing a diagnostic lumbar puncture. In contrast, serum, plasma and whole blood samples (available for DNA extraction) are collected from all patients at each visit (aim is +/-8 days from visit). All samples are stored at -80 Celsius degrees in alarmed controlled freezers. The operation and maintenance of the biobank follow the recommendations of the Swiss Academy for Medical Sciences SAMS (http://www.samw.ch/en). Peripheral blood mononuclear cells isolation may be performed as part of specific nested projects.

### Magnetic Resonance Imaging (MRI) Data

Acquisition of MRI is not mandatory within the SMSC. However, in Switzerland scans are regularly performed as part of standard care of MS patients, even in the absence of clinically evident disease activity. We aim to acquire cranial MRIs at least once a year in as many patients as possible (ideally all patients). We also aim to perform all scans within +/-28 days from the collection of clinical data and samples. A state of the art MRI protocol was agreed upon by all SMSC centres and includes: 1) High-resolution isotropic T1-MPRage (3D) without gadolinium (Gd); 2) 3D-FLAIR isotropic (if 3D acquisition not possible, 2D acquisition is accepted); 3) Axial proton density weighting (PD), 3 mm slice thickness, no gap; 4) High-resolution isotropic T1-MPRage (3D) post intravenous gadolinium (Gd) contrast administration (0.1 mmol per kg body weight, 2D acquisition is also accepted). Alignment for all axial sequences is AC/PC. Three D acquisition is performed on sagittal plane and magnet strengths of 1.5T or 3T are accepted. Advanced MRI sequences (e.g. DTI, MTR, fMRI) may be added as part of nested projects. MRI reading is performed locally and data are sent to the Medical Image Assessment Centre (MIAC) in Basel via a safe internet upload for quality control, central storage, back up and standardized analyses.

### Data Management and Quality Checks

Each patient is identified using an anonymous ID code and all individual clinical data are documented using an SMSC specific electronic Case Report Form (eCRF) developed by Rodanotech (Geneva) in collaboration with the coordinating centre. The system allows the anonymous export of data to the central database and a rapid and clear visualization of major events during the course of MS. All collected data (eCRF, MRI imaging, and banked samples) are merged at the Clinical Trial Unit (CTU) in Basel. The whole set of clinical and MRI data, as well as sampling information, is subject to several automatic and manual internal quality checks. When inconsistencies are observed (e.g. EDSS is inconsistent with functional systems scores or SPMS is diagnosed without evidence of earlier RRMS), queries are sent to the relative centre until the discrepancy is successfully solved. A standardised and updated core data set is regularly provided and made available for nested projects. This study is registered with ClinicalTrials.gov, number NCT02433028.

## Results

### Recruitment, Visits and Deviations from Protocol

**Fig 1:**
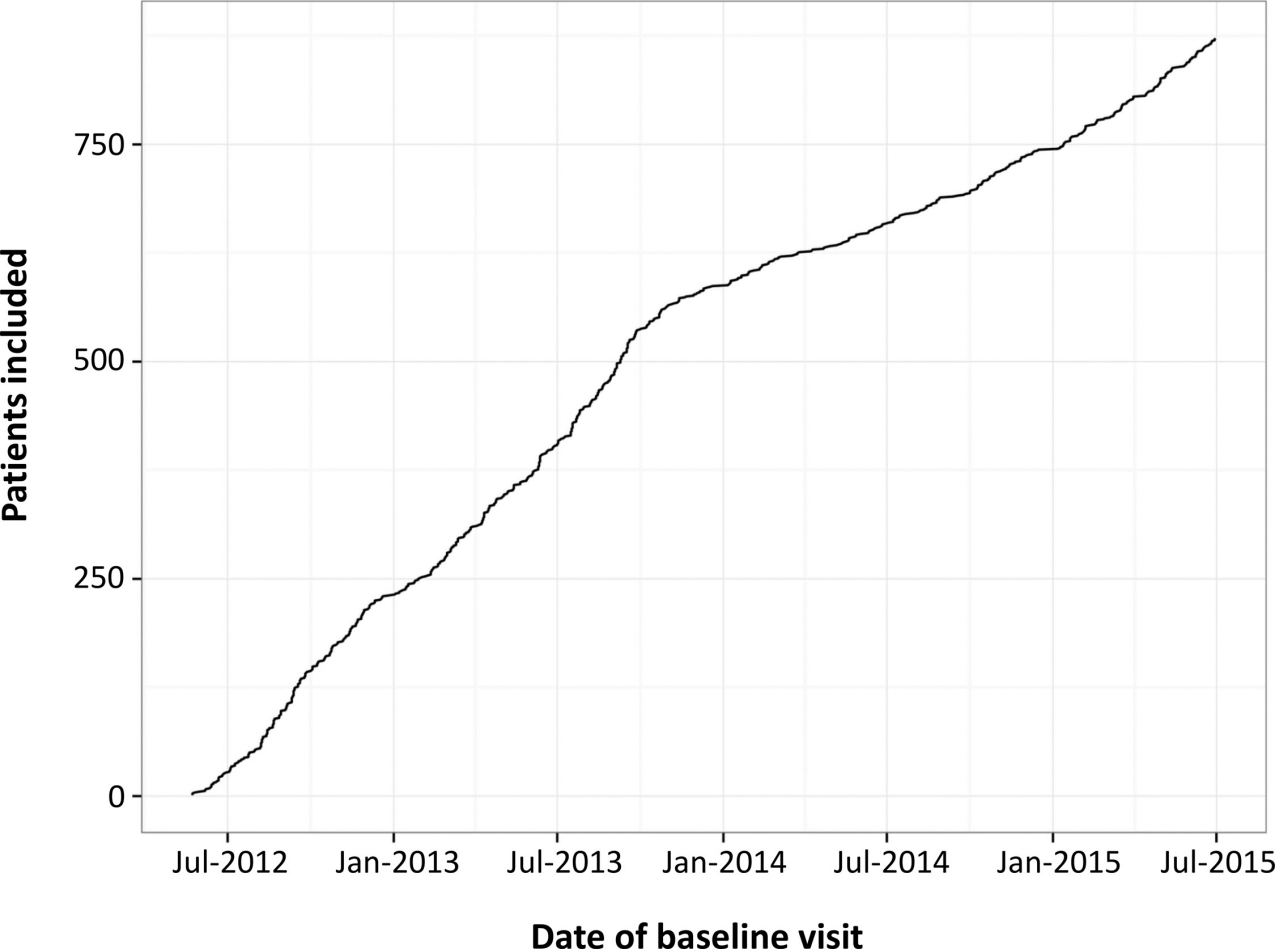
Number of recruited SMSC patients between June 2012 and July 2015.

A total of 872 patients have been recruited between June 2012 and July 2015 (Fig 1). All patients had their baseline visit and a total of 2,286 visits over a median follow-up of 398 days (interquartile range (IQR) 0–736). Only 38 patients (4.3%) have withdrawn from the study to date because of will to discontinue the study (n = 11), change of physician (n = 6), lack of response to written invitations and phone calls (n = 3), moving to a foreign country (n = 3) and other reasons (n = 11). Four deaths have occurred. Only 5 patients (0.6%) have an overdue visit (follow-up visit still missing after 12 months +/-45 days). The total number of internal quality queries has been 6,802 (99.5% successfully closed).

### Demographics of SMSC Patients

**Fig 2:**
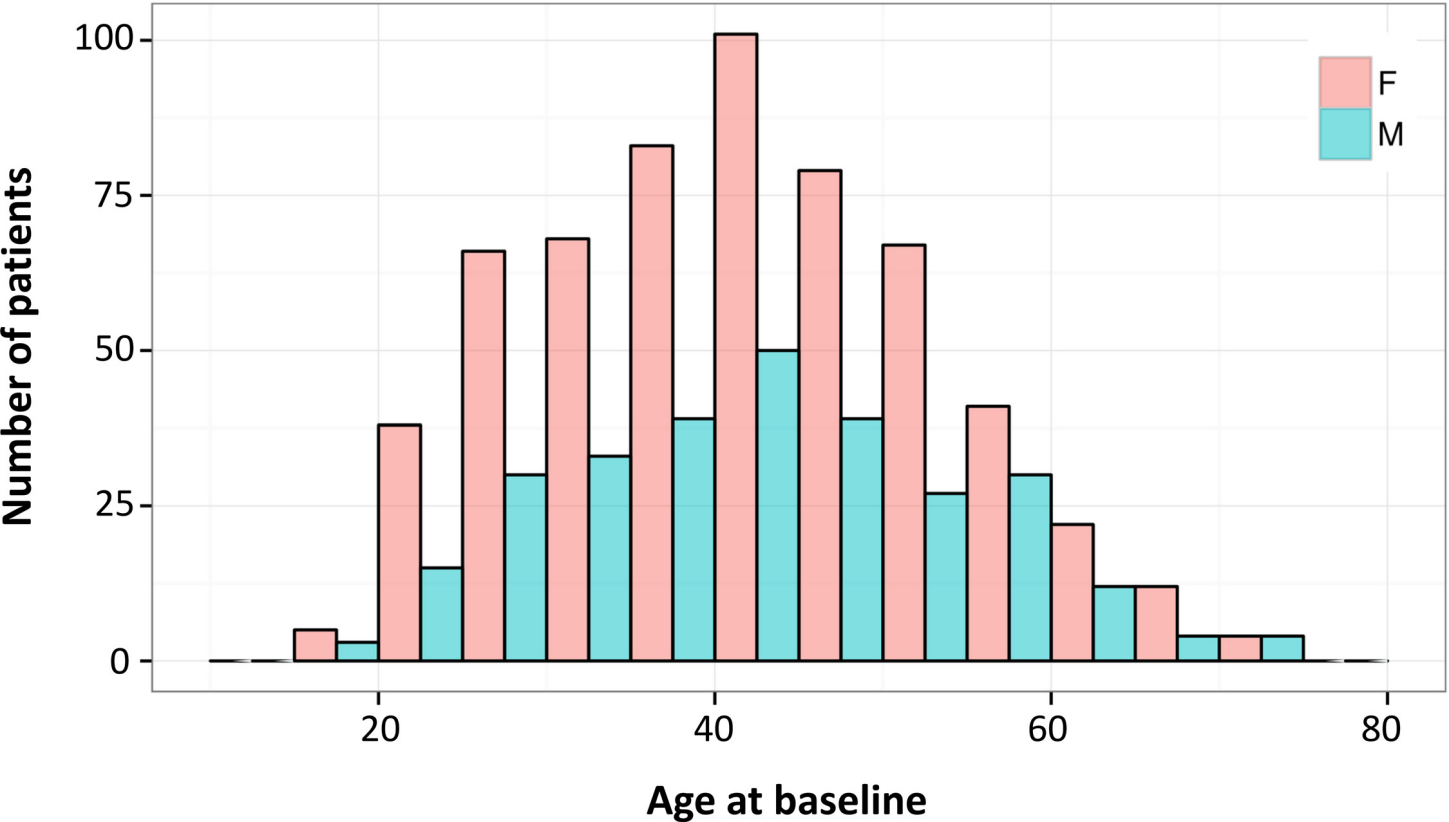
Distribution of age at baseline of all SMSC patients coloured by gender.

The total number of women and men included in the cohort at July 2015 was 586 (67.2%) and 286 (32.8%), respectively (female/male ratio = 2.0). Median age at baseline was 41.6 years (IQR 32.8–50.0) in women and 41.7 (IQR 33.6–51.3) in men (Fig 2). The vast majority of individuals were Caucasian (n = 852, 97.7%), 11 Hispanic (1.3%), 2 African (0.2%), 3 Semite (0.3%), 1 Asian (0.1%) and 3 of other origin (0.3%). The number of patients with a family history of MS was 110 (12.6%), of whom 96 had 1, 13 had 2 and 1 had 3 affected relatives.

### Baseline Clinical Features

**Fig 3:**
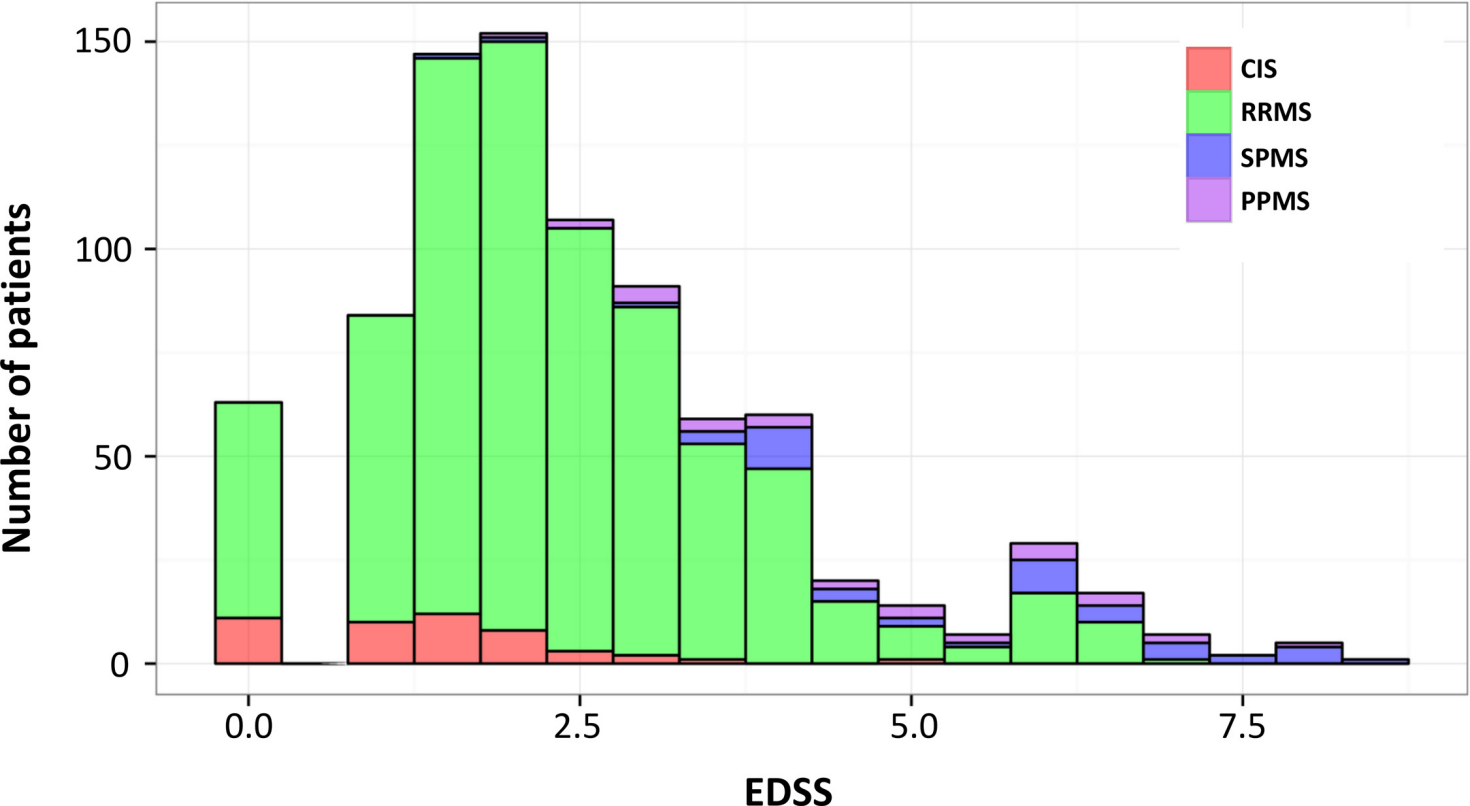
Distribution of EDSS at baseline of all SMSC patients coloured by disease course.

Baseline characteristics of all patients stratified by disease course are shown in Table 1. RRMS represented by far the largest group (85.8%), while CIS, PPMS and SPMS accounted for 5.5%, 3.5% and 5.2% of the cohort, respectively. Four RIS and 3 NMO cases have also been recruited. Age and disability scores were greater in patients with SPMS and PPMS than in CIS and RRMS. The distribution of EDSS scores stratified by disease course at baseline is presented in Fig 3.

**Table 1:** Baseline characteristics of recruited individuals stratified by diagnosis at baseline.

	CIS (n = 48)	RRMS (n = 742)	SPMS (n = 45)	PPMS (30)	RIS (n = 4)	NMO (n = 3)	All (n = 872)
**Female n (%)**	29 (60.4)	521 (70.2)	21 (46.7)	10 (33.3)	3 (75.0)	2 (66.7)	586 (67.2)
**Age median (IQR)**	36.6 (28.8–48.8)	41.0 (32.7–48.9)	54.4 (48.6–61.0)	53.4 (47.4–60.6)	43.6 (35.5–52.9)	38.4 (34.0–50.5)	41.6 (33.1–50.2)
**Days of follow-up median (IQR)**	596.0 (123.0–738.5)	385.5 (0.0–734.8)	725.0 (436.0–771.0)	378.0 (0.0–740.8)	728.0 (651.2–768.5)	828.0 (414.0–844.5)	398.0 (0.0–736.0)
**Disease duration years median (IQR)**	1.3 (0.4–3.1)	8.1 (3.2–14.0)	20.8 (13.8–29.6)	12.0 (5.2–16.3)	NA	NA	8.1 (3.1–14.4)
**EDSS median (IQR)**	1.5 (1.0–2.0)	2.0 (1.5–3.0)	6.0 (4.0–6.5)	4.8 (3.5–6.0)	NA	NA	2.0 (1.5–3.5)

**Fig 4:**
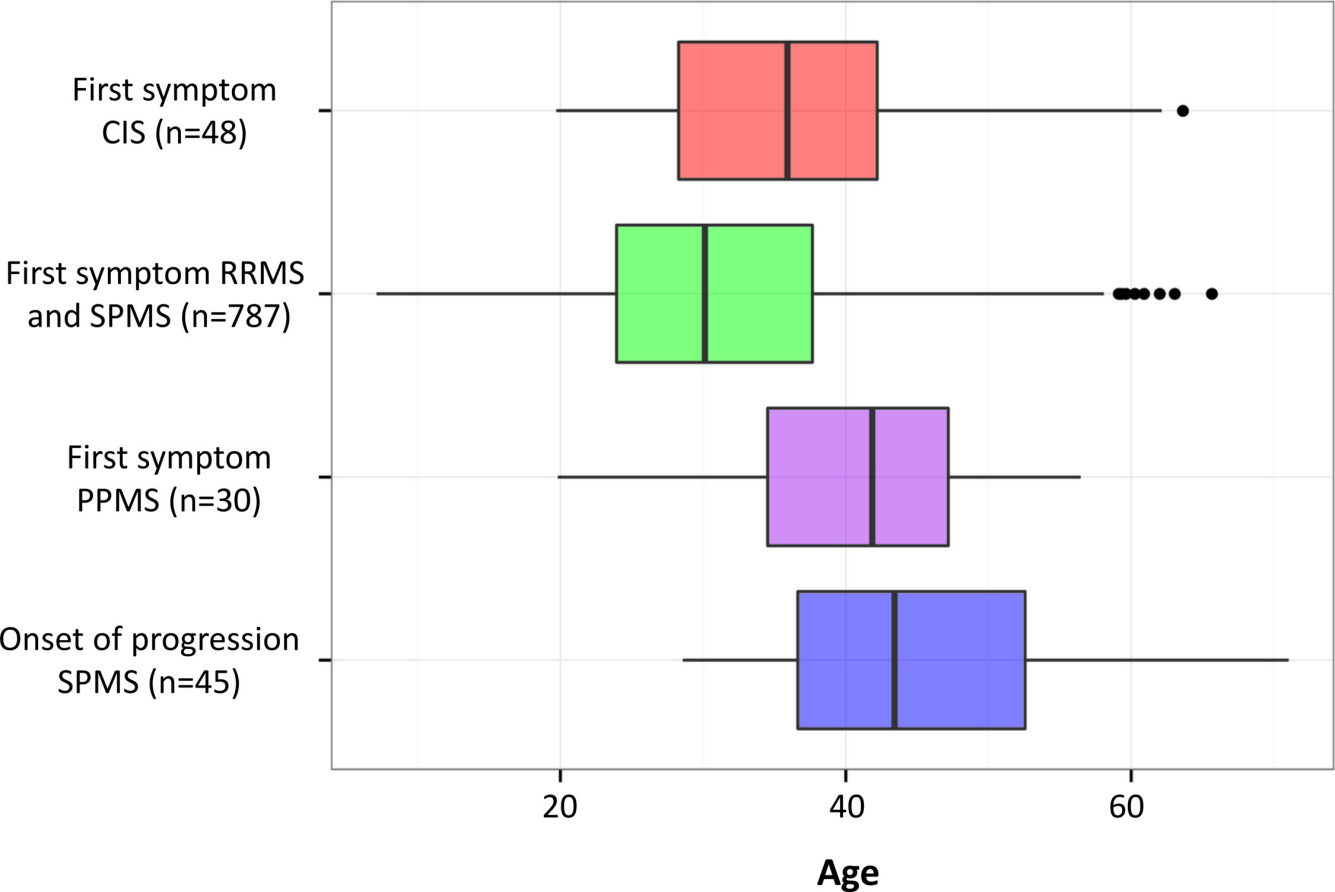
Boxplots of age at first symptom in CIS, RRMS-SPMS (combined) and PPMS patients, together with age at onset of progression in SPMS patients.

Females had their first symptom at a slightly younger age (median 30.3 years (IQR 24.2–38.3)) as compared to males (median 31.7 years (IQR 24.8–39.3). Age at first symptom appeared considerably greater in PPMS than in CIS, RRMS and SPMS patients. Age at first symptom in PPMS appeared instead similar to age at onset of progression in SPMS patients (Fig 4).

### Clinical Changes over Follow-Up

**Fig 5:**
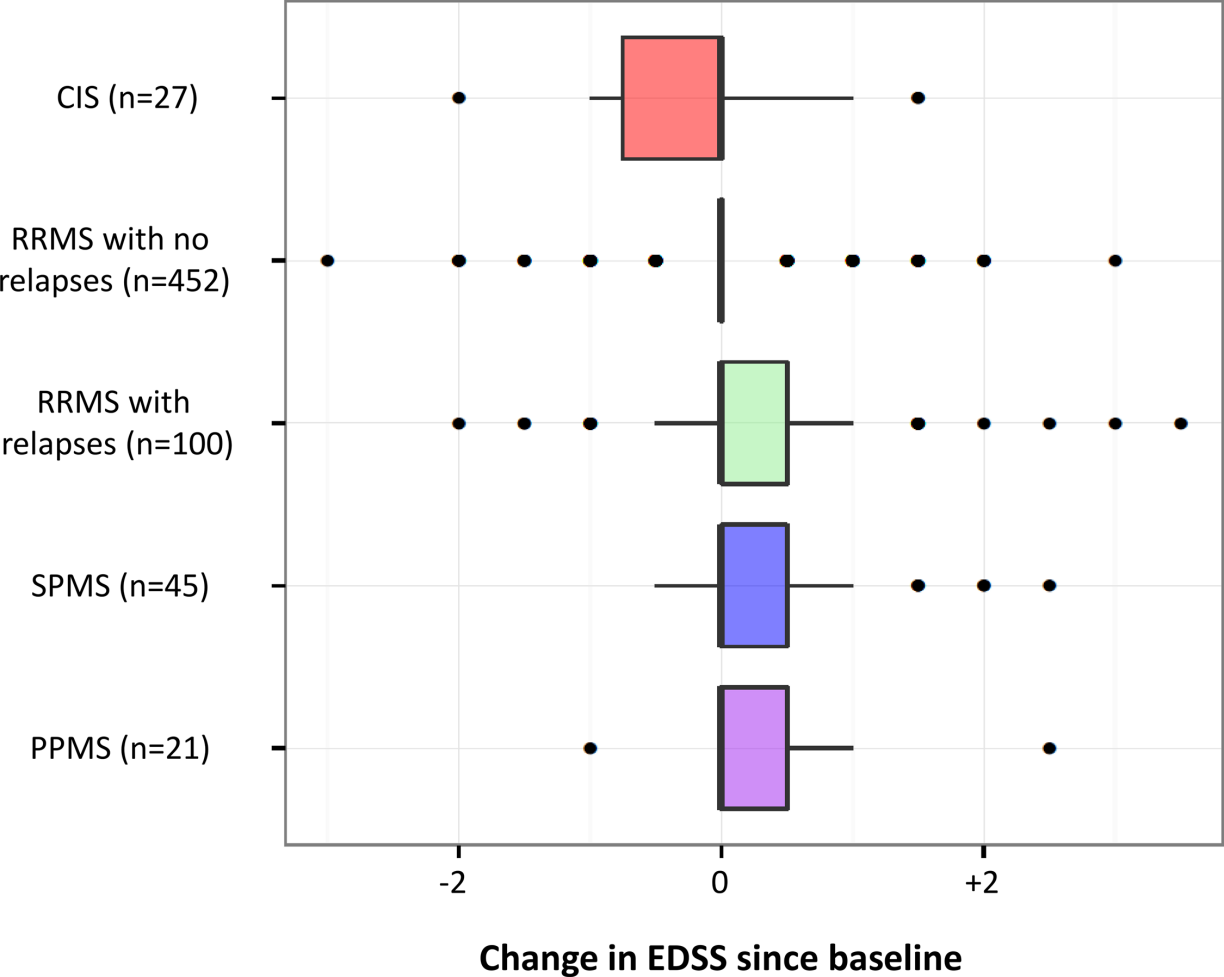
Boxplots of change in EDSS between baseline and most recent visit stratified by disease course at most recent visit and presence of relapses between baseline and most recent visit. Only patients with at least two study visits are included.

A total of 2,286 neurological examinations with EDSS assessment have been performed (90.0% by certified raters). Over the course of the follow-up, 9 CIS and 5 RRMS patients have converted to RRMS and SPMS respectively (SPMS defined as presence of progression over at least 6 months, with or without superimposed relapses preceded by an initial period of relapsing-remitting course). One, 2 and 3 relapses occurred in 122, 15 and 2 patients respectively (total number of relapses = 158). Median EDSS at most recent visits were 1.0 (IQR 1.0–1.5) in CIS, 2.0 (IQR 1.5–3.0) in RRMS, 6.0 (IQR 4.5–6.5) in SPMS and 5.2 (IQR 3.6–6.0) in PPMS. When comparing baseline vs most recent visit, trends for decreasing EDSS in CIS and increasing EDSS in RRMS patients who relapsed, PPMS and SPMS were observed. Disability in RRMS patients without relapses after baseline has instead remained overall stable (Fig 5).

### Disease Modifying Drugs

The number of individuals under treatment with specific DMDs at baseline stratified by disease course is presented in Table 2. The proportion of patients who were untreated at baseline was lower among RRMS (26.1%) than CIS (72.9%), PPMS (70.0%) and SPMS (53.3%) patients. Most RRMS patients were treated with either fingolimod (33.4%) or natalizumab (24.5%), while a smaller proportion (13.6%) was on first generation injectable DMDs. The most frequently used treatments in SPMS were injectable DMDs (24.4%), fingolimod (8.9%) and mitoxantrone (8.9%). Only 9 PPMS patients were on treatment at baseline (fingolimod n = 1, rituximab n = 1, mitoxantrone n = 1, investigational therapies n = 6).

**Table 2:** Number and percentage of patients under MS specific DMDs at baseline.

Treatments	CIS		RRMS		SPMS		PPMS		All	
	n	%	n	%	n	%	n	%	n	%
**Fingolimod**	1	2.1	248	33.4	4	8.9	1	3.3	254	29.1
**Natalizumab**	0	0.0	182	24.5	1	2.2	0	0.0	183	21.0
**Injectable DMDs**	11	22.9	101	13.6	11	24.4	0	0.0	123	14.1
**Dimethyl fumarate**	0	0.0	6	0.8	0	0.0	0	0.0	6	0.7
**Study medication**	0	0.0	2	0.3	0	0.0	6	20.0	8	0.9
**Mitoxantrone**	0	0.0	0	0.0	4	8.9	1	3.3	5	0.6
**Azathioprine**	0	0.0	2	0.3	0	0.0	0	0.0	4	0.5
**Teriflunomide**	0	0.0	3	0.4	0	0.0	0	0.0	3	0.3
**Other**	1	2.1	4	0.5	1	2.2	0	0.0	6	0.7
**Rituximab**	0	0.0	1	0.1	0	0.0	1	3.3	2	0.2
**Currently no treatment**	7	14.6	82	11.1	19	42.2	10	33.3	118	13.5
**Treatment naive**	28	58.3	111	15.0	5	11.1	11	36.7	160	18.3
**All**	48	100.0	742	100.0	45	100.0	30	100.0	872	100.0

Table 3 provides some descriptive information regarding occurrence of relapses in RRMS for the most frequently used DMDs. Data on relapses were available for all patients since 2 years before baseline visit. We calculated a treatment exposure time for DMDs that were initiated afterwards and averaged this measure across individual patients. We then calculated the number of clinical relapses, the number of patients experiencing a relapse within the exposure time and the relative annualized relapse rate (ARR, i.e. number of relapses / exposure time in years). The mean ARR progressively increased from natalizumab (0.09) to fingolimod (0.18) and injectable DMDs (0.43). Some relapses have also occurred among patients on other DMDs but sample sizes and exposure times were very small.

**Table 3:** Number of RRMS patients who initiated a DMD since 2 years before baseline, treatment exposure time, number of relapses, number of patients experiencing a relapse and annualized relapse rate (ARR). Only DMDs used in at least 10 SMSC patients and with at least 1 year of mean follow-up are shown.

Treatment	Fingolimod	Natalizumab	Injectable DMDs
**n**	343	91	80
**Days of exposure (mean (SD))**	642.5 (409.0)	639.5 (380.8)	496.7 (407.2)
**Relapses (n)**	105	17	29
**Relapsing patients (n (%))**	75 (21.9)	13 (14.3)	23 (28.7)
**ARR (mean (SD))**	0.18 (0.43)	0.09 (0.23)	0.43 (1.07)

### Biobanked Samples and MRI

A total of 2,274 serum, plasma and blood samples have been collected to date, of which 97.5% within 8 days from the date of the visit (63,378 aliquoted specimens). There were only 12 visits (0.5%) during which samples were not collected. There are 152 available CSF samples, of which 11 have been prospectively and 141 retrospectively collected. A total of 1,276 cranial MRI scans were performed, of which 85.1% within 28 days from the scheduled visit.

## Discussion

The SMSC is a prospective multicentre observational study collecting high quality clinical data, MRI scans and body fluid samples in a large group of MS patients. Almost 900 individuals were recruited between July 2012 and July 2015, for a total of 2,286 neurological examinations, 1,276 MRI scans, 2,274 blood draws and 152 collected CSF samples. The drop-out rate is extremely low. The quality of the data is ensured through several internal controls and validation steps. Careful neurological examinations are performed every 6 to 12 months and EDSS scores calculated by certified raters. The geographical characteristics of Switzerland facilitate regular visits, sustained long term follow-up and collaboration between different centres.

Patients were recruited using specific inclusion criteria and are not representative of the overall Swiss MS population. We decided to include patients who were untreated, needed to begin or switch DMD, initiated a new DMD or were currently under treatment with a second or third generation DMD. Despite being relatively broad, the application of these criteria leads to a bias in selection. In addition, as compared to the MS registries present in other countries [25–27], our sample size is inevitably smaller. Despite these limitations, we highlight the unique nature of the SMSC. Our inclusion criteria are designed to build a population of patients which is particularly suitable for analysing factors leading to disability accumulation, treatment dynamics, DMDs efficacy and safety profile. In addition, while registries mainly collect basic demographic and clinical data, the SMSC is gathering longitudinal clinical data, MRI scans and biological samples with a drop-out rate which is currently close to 0%. This would be economically unfeasible in much larger sample sizes and if all MS patients living in Switzerland were included.

We believe the current follow-up is still too short to provide meaningful results, in particular regarding disease evolution and treatment effects. We have therefore limited this manuscript to a first comprehensive description of the data without testing any statistical hypothesis. The sex ratio skewed towards females and the median age at first symptom in our cohort appear in line with current knowledge [2, 28]. We could also confirm in our cohort that the age at first symptom in PPMS is generally delayed as compared to RRMS and similar to the age at onset of progression in SPMS [29]. RRMS and PPMS patients do not differ in terms of genetic susceptibility [30] and pathological studies have not reported qualitative differences between RRMS, SPMS and PPMS [31, 32]. Taken together, these findings suggest that onset of progression is an age dependent process that occurs rather independently of relapses. Accordingly, in the latest classification of the clinical subtypes of MS, the differences between PPMS and SPMS are defined as relative and both forms belong to the spectrum of progressive disease [33].

Even in this short follow-up, disability scores have increased in SPMS, PPMS and RRMS who have relapsed since baseline. In contrast, EDSS scores tended to decrease in CIS (likely due to recovery after their first demyelinating event) and to remain stable in RRMS patients without reported relapses. These results can be considered expected, but suggest that the methods we are using to collect clinical data are appropriately describing the complex clinical course of this disease.

We have also provided some initial data regarding the use of DMDs. This is not representative of Switzerland and highly influenced by inclusion criteria and SMSC centres. We did not attempt to perform efficacy analyses at this stage, but provided some preliminary findings regarding occurrence of relapses. The relapse rate appeared lower in patients on natalizumab, intermediate in those on fingolimod and higher in those on first generation injectable DMDs. These represent relatively crude attempts to describe treatment data and we recommend extreme caution in their interpretation. Appropriate analyses will need to be performed in more homogeneous subgroups of patients using statistical methods designed for observational studies such as propensity score based tests [34]. With longer follow-up and newly recruited patients, we will also be able to investigate DMDs that have been more recently approved in MS including dimethyl fumarate, teriflunomide and alemtuzumab.

To conclude, the SMSC is a new prospective multicentre observational study designed to investigate disease evolution and new treatment options in MS. We are currently collecting high quality longitudinal clinical data in combination with biological samples and MRI data. The standardised follow-up schedule and rigorous quality control are unique features of our study compared to other MS registries. This comprehensively documented long-term cohort of patients will be an invaluable resource for rapid implementation and validation of experimental results in MS research and improved care for MS patients.
